# Specific Metabolic Fingerprint of a Dietary Exposure to a Very Low Dose of Endosulfan

**DOI:** 10.1155/2013/545802

**Published:** 2013-01-29

**Authors:** Cécile Canlet, Marie Tremblay-Franco, Roselyne Gautier, Jérôme Molina, Benjamin Métais, Florence Blas-Y Estrada, Laurence Gamet-Payrastre

**Affiliations:** INRA, TOXALIM (Research Centre in Food Toxicology), UMR 1331, INRA/INP/UPS, 31027 Toulouse, France

## Abstract

Like other persistent organochlorine pesticides, endosulfan residues have been detected in foods including fruit, vegetables, and fish. The aim of our study was to assess the impact of a dietary exposure to low doses of endosulfan from foetal development until adult age on metabolic homeostasis in mice and to identify biomarkers of exposure using an ^1^H-NMR-based metabonomic approach in various tissues and biofluids. We report in both genders an increase in plasma glucose as well as changes in levels of factors involved in the regulation of liver oxidative stress, confirming the prooxidant activities of this compound. Some metabolic changes were distinct in males and females. For example in plasma, a decrease in lipid LDL and choline content was only observed in female. Lactate levels in males were significantly increased. In conclusion, our results show that metabolic changes in liver could be linked to the onset of pathologies like diabetes and insulin resistance. Moreover from our results it appears that the NMR-based metabonomic approach could be useful for the characterization in plasma of a dietary exposure to low dose of pesticide in human.

## 1. Introduction

Many epidemiological studies have shown that exposure to pesticides is a risk factor for human health, as evidenced by the positive correlation between professional exposure to these compounds and an increase in the incidence of various human diseases (reviewed in Merhi et al. [1]). The general population is also exposed to pesticides mainly via food intake. Thus many people have a lifelong exposure to low doses of pesticides, the impact of which on human health is not yet known.

Organochlorine (OC) pesticides are among the most frequent contaminants found in the environmental compartments because they persist in the environment and bioaccumulate in organisms, partly due to their lipophilic properties [2]. Endosulfan is a chlorinated cyclodiene pesticide which was first severely restricted then banned in 2006 in several European Union countries. Nevertheless, the general population continues to be exposed: like other persistent organochlorine pesticides, endosulfan residues have been detected in several foods including fruits, vegetables, and fish [3]. There is an evidence that endosulfan is acutely poisonous to humans through both accidental and intentional exposure [4].

Endosulfan is classified by the World Health Organisation as a moderately hazardous (class II) pesticide [5]; however, it is genotoxic [6] and is an endocrine disrupter displaying xenooestrogenic activity [7]. Endosulfan has been shown to be toxic to the liver, kidney, nervous system, and reproductive organs of laboratory animals [8–10]. Exposure to this compound can modify the activity of some enzymes involved in oxidative stress and xenobiotic metabolism, as well as testosterone metabolism and clearance [11]. Recently Casabar et al. [12] showed that endosulfan was a strong activator of the pregnane X receptor (PXR) and an inducer of cytochrome P450 (CYP) 2B6 and CYP3A4, so it may have an impact on the metabolism of their substrates. A recent study by Ozmen et al. [13] showed that treating rabbits with endosulfan led to changes in glucose levels, histological degenerative changes in the pancreas and endocrine disturbances. Endosulfan may also be a risk factor for children whose parents have been exposed since the accumulation of organochlorine compounds in fat tissue during the mother's life can expose the child during pregnancy and breast feeding [2]. The transfer of OC pesticides from mother to foetus was demonstrated via the detection of the pesticides in the maternal umbilical cord, placenta, and in samples taken from the newborn [2]. Moreover, Pathak et al. [14] suggested that levels of some organochlorine pesticides such as endosulfan in the maternal fluid or tissue are associated with preterm delivery and increased foetal oxidative stress. Pre- and postnatal exposure to endosulfan is reported to affect biogenic amines and amino acids in the prefrontal cortex [15]. Whilst the toxicological effects of endosulfan have been studied *in vitro* and *in vivo*, the impact of long-term exposure to low doses through dietary intake in animal models from the foetal stage to adulthood has not yet been reported.

An NMR spectroscopy-based metabonomic approach coupled with pattern recognition technology is an effective way of characterizing the biochemical response of an organism to contaminant exposure and identifying potential biomarkers of exposure [16, 17]. Metabonomics has proven to be a very useful tool in investigating the biochemical effects of many toxic compounds [18–20].

In our study we developed a mouse model of diet to assess the impact of dietary exposure to a low dose of endosulfan on metabolic homeostasis in mice exposed from foetal development until adult age. We investigated biomarkers of dietary exposure using an ^1^H-NMR-based metabonomic approach in various tissues and biofluids.

## 2. Materials and Methods

### 2.1. Reagents

Endosulfan was purchased from Fluka (Riedel de Haën, France) and was added as a component of rodent nuggets at a dose of 30 *μ*g per kg. This dose allowed the mice to ingest the equivalent of the Acceptable Daily Intake (ADI 0.006 mg/kg food/day)) defined for humans by the Joint FAO/WHO Meeting on Pesticide Residues [21] that was extrapolated for mice on the basis of mean body weight. Information on the toxicity of endosulfan was obtained from Agritox (AFSSA, 2005. http://www.dive.afssa.fr/agritox/) or ExToxNet (ExToxNet: http://extoxnet.orst.edu/).

### 2.2. Preparation of Pesticide-Enriched Feed

Endosulfan (1.5 mg) was dissolved in 1 ml methanol, and then 100 *μ*L of this solution (containing 150 *μ*g endosulfan) was mixed with methanol (final quantity of endosulfan 150 *μ*g). The whole resulting solution (containing 150 *μ*g endosulfan) was mixed with acetone (9 mL) and dispersed on 10 g of the vitamin mixture powder (vitaminic mixture 200, Scientific animal Food Engineering (Safe), France). The vitamin powder containing 150 *μ*g endosulfan was then homogenized in a rotavapor for 30 minutes at 45°C to evaporate the solvent and then mixed for 50 minutes at room temperature with the remaining quantity of vitamin mixture (40 g) needed to make the rodent nuggets (5 kg). The vitamin powder enriched with endosulfan was then sent to the INRA animal feed preparation unit (UPAE INRA Paris France) where vitamins (1%) and mineral complement (7%) were mixed with the other components of the rodent nuggets. The presence of endosulfan was quantified by Eurofins (Nantes, France). The final quantity was around 30 *μ*g/kg of food. Control feed was prepared as described above with a mixture of methanol/acetone (1/1, V/V) but lacking pesticide. Control feed was analyzed for the presence of the endogenous pesticide (endosulfan) and also for the presence of the most common pesticides found in the environment (including the organophosphorus pesticide organochloride, pyrethroid, and PCB). Results showed that nuggets in control food do not contain pesticides at the detectable level (0.01 mg/kg).

### 2.3. Animals

Ten-week-old female and male C57 BL/6J mice were purchased from Charles River Laboratories, France. Mean body weights were 20 and 24 g for females and males, respectively. Females (5 per lot) were fed from mating until gestation and lactation with the control diet or the diet enriched with pesticide. Weaned pups (from 6 to 8 per lot) were then fed with the same diet as their respective parent for an additional period of 11 weeks. Food consumption was monitored and the body weight was measured. Animals were sacrificed by cervical dislocation, and the spleen, liver, and kidneys were weighed. Bone marrow was extracted from the femur of treated or nontreated males and females as previously described [1, 22]. The liver was excised, weighed, and frozen rapidly in liquid nitrogen. In addition, blood samples were taken from the facial artery of each animal and added to a glass vial containing heparin (Multivette, SARSTED, Germany).

### 2.4. Metabonomic Studies

#### 2.4.1. Sample Preparation for ^1^H NMR Spectroscopy

Plasma samples (100 *μ*L) were diluted with 500 *μ*L deuterium oxide (D_2_O) before being placed in 5 mm NMR tubes.

Bone marrow cells were mixed with 600 *μ*L D_2_O then sonicated in an ice bath for 1 min. Samples were centrifuged for 10 min at 4°C at 5000 g, and the supernatants were transferred to NMR tubes.

For liver samples the organ extraction method used here, which derives from the Folch procedure [23], was adapted from the method described by Waters et al. [24]. Samples of liver tissue (~100 mg) were homogenized with a Polytron PT 2100 in 2 mL acetonitrile/H_2_O (50/50, v/v) containing 0.1% butylated hydroxytoluene (BHT) in an ice-water bath. The homogenates were centrifuged for 10 min at 4°C at 5000 g, and the supernatants were removed and lyophilised. The resulting powders were reconstituted in 600 *μ*L of deuterium oxide (D_2_O). The reconstituted solutions were transferred to NMR tubes.

#### 2.4.2. ^1^H Nuclear Magnetic Resonance (NMR) Analyses

All ^1^H NMR spectra were obtained on a Bruker DRX-600 Avance NMR spectrometer operating at 600.13 MHz for ^1^H resonance frequency using an inverse detection 5 mm ^1^H-^13^C-^15^N cryoprobe attached to a CryoPlatform (the preamplifier cooling unit).

The ^1^H NMR spectra of plasma and bone marrow samples were acquired at 300 K using the Carr-Purcell-Meiboom-Gill (CPMG) spin-echo pulse sequence with presaturation with a total spin-echo delay (2n*τ*) of 40 ms to attenuate broad signals from proteins and lipoproteins. A total of 128 transients were acquired in 32 K data points using a spectral width of 12 ppm and an acquisition time of 2.28 s. Prior to Fourier transformation, an exponential line broadening function of 0.3 Hz was applied to the FID.

The ^1^H NMR spectra of liver extracts were acquired at 300 K using the standard ^1^H pulse sequence, accumulating 128 free induction decays into 32 K data points with a relaxation delay of 2 s. A 12 ppm spectral width was used. The data were apodized with an exponential function using a line broadening of 0.3 Hz prior to Fourier transformation. To confirm the chemical structure of the metabolites of interest, 2D ^1^H-^1^H COSY (Correlation Spectroscopy) and 2D ^1^H-^13^C HSQC (Heteronuclear Single Quantum Coherence Spectroscopy) NMR experiments were performed on selected samples. For the ^1^H-H COSY NMR experiment, a total of 32 transients were acquired in 4096 data points. A total of 256 increments were measured in F1 using a spectral width of 10 ppm and an acquisition time of 0.28 s. The data were weighted using a sine-bell function in *t*_1_ and *t*_2_ prior to Fourier transformation (FT). ^1^H-^13^C HSQC NMR spectra were collected in selected samples with ^1^H detection. A relaxation delay of 2.5 s was used between pulses, with a refocusing delay equal to 1/4^1^ *J*_C−H_ (1.78 ms). A total of 2048 data points with 64 scans per increment and 512 experiments were acquired with spectral widths of 10 ppm in F2 and 180 ppm in F1. The data were multiplied by a shifted Qsine-bell function prior to FT.

Spectral assignment was based on matching 1D and 2D data to reference spectra in a home-made reference database, as well as with other databases (http://www.bmrb.wisc.edu/ and http://www.hmdb.ca/), and reports in the literature.

#### 2.4.3. Data Reduction and Multivariate Statistical Analyses

All NMR spectra were phased and the baseline corrected. The data was then reduced using AMIX (version 3.8, Bruker, Analytik) to integrate 0.04 ppm wide regions corresponding to the *δ* 10.0–0.5 ppm region. The *δ* 5.1–4.5 ppm region, which includes the water resonance, was excluded. To account for differences in sample amounts, each integrated region was normalized to the total spectral area. Multidimensional statistical analyses of NMR data were performed using Simca-P11 software (Umetrics, Umeå, Sweden). Principal components analysis (PCA) was applied to the pareto-scaled spectral data to reveal treatment-related patterns. Projection to latent structure discriminant analysis (PLS-DA) was also performed to improve the classification of the different groups of mice. Dummy variables, containing 0 and 1, were created to describe the class membership of each observation. The number of components in the PLS models was chosen by cross-validation (7-fold). The parameters *R* ^2^ and *Q* ^2^ were used as measures for the robustness of a pattern recognition model. *R* ^2^ is the fraction of variance explained by a component, and cross-validation of *R* ^2^ gives *Q* ^2^ which reveals the fraction of the total variation predicted by a component; it is a measure of the difference between two (or more) groups. Typically a robust model has *R* ^2^ > 50% and *Q* ^2^ > 0.4.

In addition, the statistical significance and validity of the PLS-DA results were assessed using a permutation test. This test can determine whether the specific classification of individuals in the designated groups is significantly better than any other random classification in two arbitrary groups [25]. In this procedure, the *Y* matrix (group membership) is randomly permuted a number of times (200 times in this study), while the *X* matrix (NMR buckets) is kept unchanged. For each “permuted” sample, a PLS-DA model is constructed, the *R* ^²^/*Q* ^²^ values calculated and compared to the values of the “original” model. As a result of this test, *R* ^²^ and *Q* ^²^ values of the “original” model (on the right of biplot) and the “permuted” models are displayed versus the correlation between the “original” *Y* matrix and the “permuted” *Y* matrix. A regression line is estimated from this biplot. Valid models correspond to a negative *Q* ^²^ intercept of the regression line.

Data were preprocessed using Orthogonal Signal Correction (OSC) with the treatment as a correction factor. The OSC filtering [26] was applied to remove variation not linked to the treatment (confounding factors such as physiological, experimental, or instrumental variation).

The identification of major metabolic perturbations within the pattern recognition models was achieved by analysis of corresponding loading plots and variable importance in the projection (VIP). VIP, a global indicator measuring the influence of each variable on the PLS components, was used to derive a subset of the most important variables for the separation of experimental groups. An arbitrary threshold of VIP >1.0 was chosen to select the variables.

Imported data were mean centered and Pareto scaled for all PLS analyses.

Univariate statistical tests using *R* software were also carried out on the bucket integrated NMR spectra, comparing endosulfan-treated to control mice for bone marrow, plasma and liver samples. Each bucket was treated as an independent variable. Statistically significant changes between the distributions of treated animals and the control group were assessed using a parametric Student's *t*-test as well as the nonparametric Kruskal-Wallis test. A value of *P* < 0.05 was considered significant. The software *R* and the packages used in this work were downloaded freely from the website (http://www.r-project.org/).

## 3. Results

Throughout our investigation we found no difference in body weight between endosulfan-treated and control groups, and there was no difference in the weight of the liver, or brain between the groups (data not shown). However a slight but significant decrease was observed in kidney in females.

To identify the metabolic fingerprint characterizing dietary exposure to endosulfan at low doses, high-resolution ^1^H NMR spectra were recorded from samples of bone marrow, plasma, and liver of control and endosulfan-treated 14-week-old mice. Thirty-one metabolites were identified, and Table 1 lists the compounds assigned based on their 1D and 2D spectra. From these results, PLS-DA analysis of each tissue was carried out as described in the materials and methods section; this highlighted the different effects of treatment between male and female mice. Since the metabolic profiles of each gender are quite distinct, female and male mice were considered independently in order to identify the metabolic changes resulting from endosulfan treatment. Intercept values of permutation test, showing the robustness of the models, are presented in Table 2 for PLS-DA models per sex. All metabolic changes are summarized in Table 3.

### 3.1. Metabolic Profiles of Plasma from Endosulfan-Treated Mice

**Figure 1:**
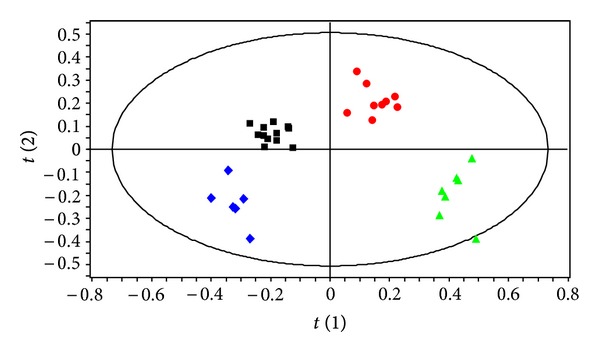
PLS-DA score plot based on the ^1^H NMR spectra of plasma samples from nonexposed males(green triangle, *n* = 7),exposed male (red circle, *n* = 9), nonexposed females (blue diamond, *n* = 6), and exposed female mice (black square, *n* = 12) (*Q* ^2^ = 0.60, *R* ^2^ = 72.7%, and A = 3 (Latent Variables fitted)).

**Figure 2:**
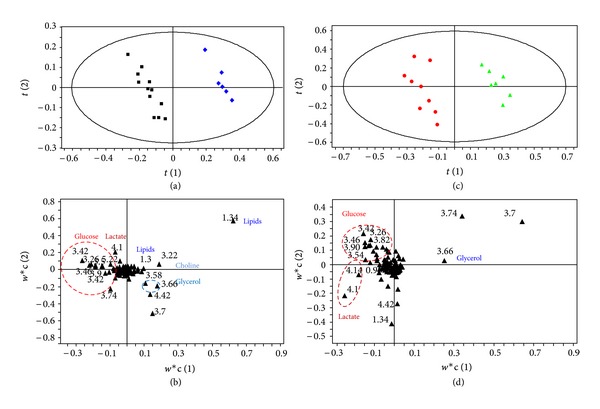
PLS-DA (a) score plot and (b) loading plot based on the ^1^H NMR spectra of plasma samples from nonexposed (blue diamond, *n* = 6) and exposed female mice (black square, *n* = 12) (*Q* ^2^ = 0.95, *R* ^2^ = 98.9%, and A = 2). PLS-DA (c) score plot and (d) loading plot based on the ^1^H NMR spectra of plasma samples from nonexposed (green triangle, *n* = 7) and exposed males (red circle, *n* = 9) (*Q* ^2^ = 0.83, *R* ^2^ = 97.6%, and A = 3).

Pattern recognition of the ^1^H NMR spectra from plasma of mice exposed to dietary endosulfan is shown in Figure 1. The PLS-DA score plots clearly distinguish samples of exposed mice from those of nonexposed mice along PLS-DA component 2. Animal gender is also separated along PLS-DA component 1. PLS-DA models were built to model the differences between endosulfan-exposed males separately to those of females (Figures 2(a) and 2(c)). These models were robust and predictable (*R* ^2^ = 0.989 and *Q* ^2^ = 0.95 for females and *R* ^2^ = 0.976 and *Q* ^2^ = 0.83 for males). Permutation tests showed that the model was not overfitted (Table 2). Loading plots in Figures 2(b) and 2(d) show discriminant metabolites in treated and untreated animals, in females and males, respectively. Discriminant metabolites were summarized in Table 3. Dietary exposure of female mice to endosulfan led to a decrease in plasma LDL and/or VLDL. Biochemical analysis of female plasma confirmed the decrease in LDL level (data not shown). Dietary exposure of females to endosulfan also led to a significant decrease in choline levels and a clear increase in glucose levels. The categories of metabolites identified in plasma samples from exposed male mice were different to those of female mice except the change in glucose levels which were also observed in both genders. Lactate levels were increased in males exposed to endosulfan but no changes in choline or VLDL and/or LDL levels were observed.

### 3.2. Metabolic Profiles of Bone Marrow from Endosulfan-Treated Mice

**Figure 3:**
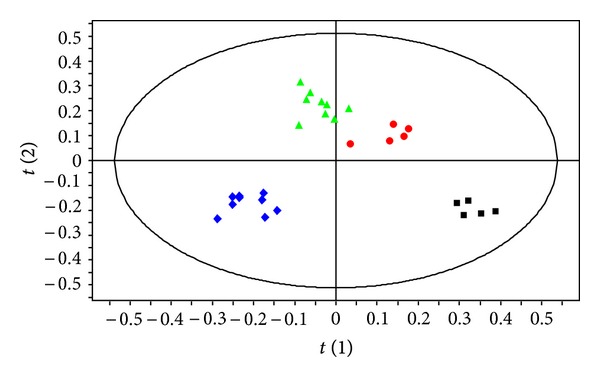
PLS-DA score plot based on the ^1^H NMR spectra of bone marrow samples from nonexposed male (green triangle, *n* = 7), exposed male (red circle, *n* = 5), nonexposed female (blue diamond, *n* = 9) and exposed female mice (black square, *n* = 5) (*Q* ^2^ = 0.94, *R* ^2^ = 97.5%, and A = 4).

**Figure 4:**
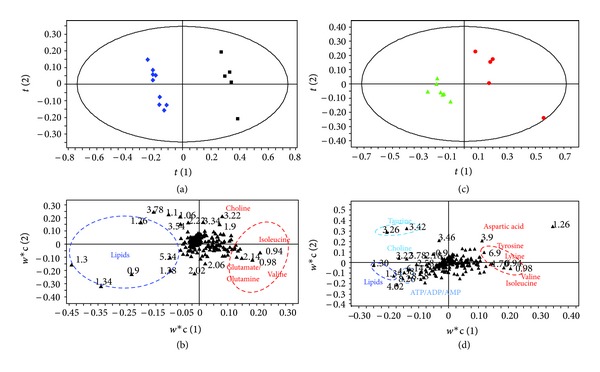
PLS-DA (a) score plot and (b) loading plot based on the ^1^H NMR spectra of bone marrow samples from nonexposed (blue diamond, *n* = 9) and exposed female mice (black square, *n* = 5) (*Q* ^2^ = 0.92, *R* ^2^ = 99.6%, and A = 2). PLS-DA (c) score plot and (d) loading plot based on the ^1^H NMR spectra of bone marrow samples from nonexposed (green triangle, *n* = 7) or exposed male (red circle, *n* = 5) (*Q* ^2^ = 0.97, *R* ^2^ = 99.5%, and A = 4).

The PLS-DA model, described in the materials and methods section, separated all four groups of bone marrow samples as shown in Figure 3 (*R* ^2^ = 0.975 and *Q* ^2^ = 0.94). Bone marrow from endosulfan-exposed and nonexposed mice appear as distinct metabolic groups along PLS-DA component 1, whereas animal gender appears separately along PLS-DA component 2. To further clarify the impact of treatment in the sample groups of each gender, other PLS-DA models were built, and the results modelled separately for male and female sample groups as shown in Figures 4(a) and 4(c). For both gender groups the ^1^H NMR profiles derived from endosulfan-treated mouse samples were distinguished from those of the controls using PLS-DA analysis. These models were robust and predictable (*R* ^2^ = 0.996 and *Q* ^2^ = 0.92 for females and *R* ^2^ = 0.995 and *Q* ^2^ = 0.97 for males). Permutation tests showed that the model was not overfitted (Table 2). Loading plots in Figures 4(b) and 4(d) show discriminant metabolites in treated and untreated animals in females and males, respectively. Discriminant metabolites were summarized in Table 3. For both female and male mice dietary exposure to endosulfan resulted in a decrease in fatty acids in bone marrow, characterized by the long chain lipid (CH_2_)_*n*_ signal and by an increase in valine and isoleucine. Gender differences were clearly observed: in treated females the levels of lysine, alanine, succinate, and glutamate/glutamine were increased; in contrast male mice had increased levels of acetate, tyrosine, and inosine upon exposure to endosulfan whereas the level of lactate and ATP/ADP/AMP decreased. The level of choline compounds was shown to be increased in females and decreased in males.

### 3.3. Metabolic Profiles of Aqueous Liver Extracts from Endosulfan-Treated Mice

**Figure 5:**
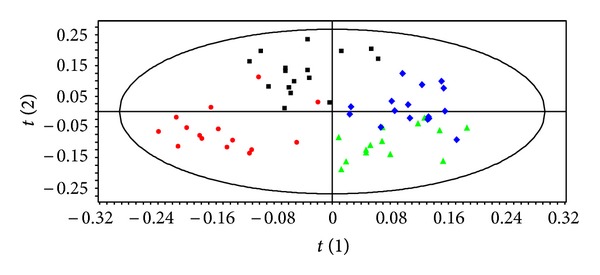
PLS-DA score plot based on the ^1^H NMR spectra of aqueous liver extracts sampled from nonexposed male (green triangle, *n* = 14), exposed male (red circle, *n* = 15), nonexposed female (blue diamond, *n* = 16) and exposed female mice (black square, *n* = 16) (*Q* ^2^ = 0.657, *R* ^2^ = 73.3%, and A = 3).

**Figure 6:**
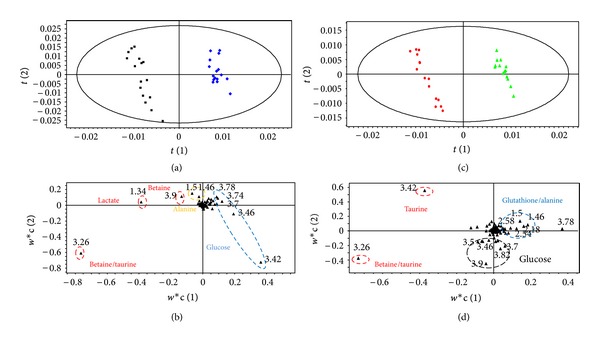
PLS-DA (a) score plot and (b) loading plot based on the ^1^H NMR spectra of aqueous liver extract samples from nonexposed (blue diamond, *n* = 16) and exposed female mice (black square, *n* = 16) (*Q* ^2^ = 0.965, *R* ^2^ = 98.4%, and A = 2). PLS-DA (c) score plot and (d) loading plot based on the ^1^H NMR spectra of aqueous liver extract samples from nonexposed (green triangle, *n* = 14) or exposed male (red circle, *n* = 15) (*Q* ^2^ = 0.93, *R* ^2^ = 99.1%, and A = 3).

The PLS-DA score plots of liver samples clearly separated exposed mice from nonexposed mice along PLS-DA component 1 (Figure 5). Gender was separated along PLS-DA component 2. For the control group, the distinction between male and female mice was not clear. PLS-DA models were then built to model the differences between exposed males and females separately (Figures 6(a) and 6(c)). These models were robust and predictable (*R* ^2^ = 0.984 and *Q* ^2^ = 0.965 for females and *R* ^2^ = 0.991 and *Q* ^2^ = 0.93 for males). Permutation tests showed that the model was not overfitted (Table 2). Loading plots in Figures 6(b) and 6(d) show discriminant metabolites in treated and untreated animals, in females and males respectively. Discriminant metabolites were summarized in Table 3. In males and females, hepatic metabolic disturbances were characterized by a net decrease-oxidized glutathione concomitantly with an increase of taurine and betaine levels, suggesting a perturbation of oxidative status upon endosulfan exposure. In females glucose levels were decreased whereas lactate levels were increased. By contrast in male mice dietary exposed to endosulfan, no changes were observed in glucose level whereas lactate level was decreased. In addition, in male mice, 5 metabolites were found to distinguish from females: alanine, glutamate/glutamine, and glycogen whose levels decreased, and choline whose level increased.

## 4. Discussion

Endosulfan is an organochlorine pesticide which was banned in France in 2006. However, recent EFSA reports have shown that residues of this compound are still found in some fruits and vegetables in the European Union [3]. In addition, due to its capacity to bioaccumulate, endosulfan is still found in human biological fluids and tissues. Subchronic exposure to this contaminant commonly affects the central nervous system [15, 27], immune [28], and reproductive systems [29]. These effects have been at least partly linked to an increase in oxidative stress [30–32]. Occupational exposure to pesticides is often linked to an increased risk of developing certain pathologies in adults and children [1, 33]. The general population is also exposed to pesticides mainly through the presence of contaminants in food, where consumers are exposed to low doses of pesticides alone or in mixtures throughout their life. Although consumer exposure is different from occupational exposure in mode, quantity, duration, frequency, and so forth, improved knowledge of the influence of such exposure on health is needed. Moreover, since exposure of the general population to pesticides cannot be quantified, the development of approaches to enable identification of biological markers of exposure to these compounds is of great interest for epidemiological studies.

In this study, we investigated the metabolic fingerprint of a dietary exposure to low doses of endosulfan using an ^1^H NMR-based metabonomic approach on bone marrow, liver, and plasma in male and female mice. Our results show that dietary exposure to endosulfan is characterized by specific metabolic fingerprints in plasma, liver, and bone marrow of mice exposed to dietary endosulfan. The PLS-DA models obtained in this study are valid and robust, as shown by cross-validated *Q* ^²^ values and *R* ^²^ and *Q* ^²^ intercept values of permutation test (Table 2). The main changes observed were an increase in plasma glucose in both gender, a decrease in LDL levels especially in females and biochemical changes to the membrane composition of bone marrow cells (lipids and choline/phosphocholine). The variations in taurine, betaine, and oxidized glutathione in both genders could be linked to a disturbance of oxidative status upon endosulfan exposure; glucose, lactate variations could be useful as biomarkers for liver function changes in both males and females. Some metabolic changes appear to be gender-specific (e.g., plasma lipids LDL and hepatic levels of lactate and glucose).

### 4.1. Endosulfan-Induced Disturbances in Energy Metabolism

Changes to the level of metabolites involved in energy metabolism were observed upon dietary exposure to low doses of endosulfan during the pre- and postnatal period. Dietary exposure of female mice to endosulfan led to both an increase in plasma glycaemia and a decrease in endogenous hepatic glucose. The altered glucose levels could be related to the induction of the glycolytic pathway since a concomitant increase in liver lactate levels was also observed in females upon exposure. In contrast, in male mice the increase in plasma glucose levels appeared to be more associated with a decrease in activity of the hepatic glycolytic pathway, since lactate levels were decreased in the liver. Insulin insufficiency could also be causing the elevated blood glucose levels observed in exposed male and female mice. Indeed, endosulfan has already been shown to affect glucose levels in rat plasma and to have an impact on the B cells of the pancreas that are responsible for insulin secretion [13, 34]. Although it cannot be confirmed from the data presented here, our results are in agreement with dysfunctional insulin-mediated regulation of glucose utilisation by tissues. Moreover our data showed that, even at low doses, endosulfan caused changes to endogenous glucose content in the liver and plasma levels that could be linked to the onset of pathologies such as diabetes or insulin resistance. A physiological response to such hyperglycaemia could increase the consumption of glucose by muscle and fat tissue. This needs to be confirmed by further experiments. Our results are also in agreement with Lee et al., who observed that serum concentrations of OC pesticides are positively and significantly correlated with high fasting glucose in a population of nondiabetic human subjects [35].

Taurine levels in male mice were increased in liver upon endosulfan exposure. Taurine is an important metabolite involved in bile acid synthesis, osmoregulation, and intracellular calcium levels [36]. It is generally regarded as a sensitive marker indicating changes in liver metabolism. Furthermore, betaine levels were significantly increased in the liver of both genders. Betaine and taurine are important osmolytes and appeared to be critical for proper liver function [36]. Osmolytes have been shown to affect protein stability against a wide variety of adverse environmental conditions [37]. The increase of betaine or taurine could be linked to hepatic toxicity, in the same way as the increase in creatine in females could reflect liver physiopathological change.

### 4.2. Endosulfan-Induced Changes in Choline Metabolism

Choline is synthesized by the liver. It plays an important role in the integrity and composition of cell membranes. Phosphocholine is the most abundant phospholipid in biological membranes and, together with other lipids, forms the characteristic bilayer structure of cells and regulates membrane integrity. Our results suggest that dietary exposure to low doses of endosulfan could lead to changes of haematopoietic cell membrane composition. Changes to the biochemistry of membrane lipids after treatment with endosulfan have also been observed by other authors [38, 39]. Changes to the membrane composition of bone marrow cells could have a major impact on the proliferative activity of haematopoietic cells. The increased choline content may be also linked to increased cell division since choline values have been shown to be predictive of proliferative activity of glioma [40–42]. This point is currently under investigation in our lab.

At the plasma level, as choline is the main component of VLDL, the decreased level of choline in females upon exposure to endosulfan could be mediating the decrease of this plasma lipoprotein. Biochemical analysis of plasma also showed a significant increase in triglyceride content that could be linked with changes in choline and VLDL levels. This result is in agreement with the clear decrease of plasmatic glycerol levels in both genders since this metabolite is an indirect indicator of TG hydrolysis.

Choline is also a precursor for the synthesis of acetylcholine whose synthesis depends on the capture of choline in the blood. The level of circulating choline could have an impact at the neuronal level by accelerating neuronal transmission. This could be compared with the neurotoxicity of endosulfan which has been described elsewhere [43].

Taken together, these results suggest that dietary exposure to endosulfan even at low doses could interfere with a number of physiological processes that regulate cell division, lipid metabolism, and neuronal transmission.

### 4.3. Endosulfan-Induced Disturbance in Oxidative Stress Metabolism

The levels of oxidized glutathione in the liver were decreased in both males and females upon dietary exposure to endosulfan, suggesting a cellular response linked to oxidative stress, although it cannot be confirmed from the presented data. One the other hand no differences in reduced glutathione level have been observed between endosulfan exposed- and control group. Unchanged reduced glutathione levels between the two groups could be due to an efficient GSH generation in endosulfan-exposed animals through the increase of betaine level (trimethylglycine). Indeed betaine is a primary methyl donor for S-adenosylmethionine which is known to regulate glutathione concentrations under conditions of oxidative stress in the liver [44].

The decrease in oxidized glutathione levels in the liver of male mice was associated with a concomitant decrease in glutamine and glutamate in the liver upon endosulfan exposure. Glutamate is a precursor for synthesis of glutathione and in this way forms a part of the key antioxidant system. Glutamine and glutamate cycle back and forth, converting from one form to the other during normal body metabolism. Our results are in agreement with reports that endosulfan induces oxidative stress, particularly in male mice [32, 38, 45], and studies are under investigation to confirm this hypothesis.

As a fuel for rapidly dividing cells, glutamine contributes towards a healthy immune system, especially in the rapid production of white blood cells during an infection. The increased level of glutamine in female mouse bone marrow upon endosulfan exposure could be linked with its impact on the immune system [46].

## 5. Conclusion

Our metabonomic approach has enabled the detection of metabolic disturbances following dietary exposure to a low dose of endosulfan. Here we show that these disturbances are associated with oxidative stress in the liver as well as changes in hepatic glucose metabolism.

Given the increase in plasma glucose levels following endosulfan exposure, we believe that metabonomics will prove to be a useful approach to characterize dietary exposure in human. These noninvasive methods will be highly valuable tools for assessing the real exposure to pesticides in epidemiological studies.

## Figures and Tables

**Table 1:** ^
1^H and ^13^C resonance assignments with chemical shifts, multiplicity, and *J*-couplings for signals identified in plasma, bone marrow, and liver in mice.

Compound	*δ*_H_ ppm (multiplicity, coupling constant, assignment)/*δ*_C_ ppm	Biological matrice
Acetate	1.92 (s, CH_3_)/25.9	Liver, bone marrow
Alanine	1.48 (d, *J* = 7.1 Hz, CH_3_)/18.9; 3.78 (q, *J* = 7.2 Hz, CH)/53.1	Liver, plasma
ATP/ADP/AMP	4.51 (m, CH_2_)/73.3; 6.15 (d, *J* = 5.9 Hz, CH)/89.5; 8.27 (s, CH, ring)/155.2; 8.58 (s, CH, ring)/142.6	Liver, bone marrow
Betaine	3.27 (s, CH_3_)/56.1; 3.91 (s, CH_2_)/68.9;	Liver
Choline	3.22 (s, N(CH_3_)_3_)/56.9; 3.52 (m, NCH_2_)/70.0; 4.05 (m, CH_2_)/58.3	Liver, plasma, bone marrow
Citrate	2.54 (d, *J* = 15 Hz, 2CH)/48.7; 2.70 (d, *J* = 15 Hz, 2CH)/48.7	Plasma, bone marrow
Creatine	3.93 (s, CH_2_)/56.4; 3.03 (s, CH_3_)/39.9	Liver, plasma, bone marrow
*α*-Glucose	3.41 (m, CH)/72.3; 3.54 (m, CH)/73.9; 3.71 (m, CH)/75.4; 3.83 (m, CH)/74.2; 3.84 (m, CH)/63.3; 5.23 (d, CH, *J* = 3.8 Hz)/94.8	Liver, plasma
*β*-Glucose	3.25 (m, CH)/76.9; 3.41 (m, CH)/72.3; 3.48 (m, CH)/78.4; 3.73 (m, CH)/63.3; 3.90 (m, CH)/63.4; 4.64 (d, CH, *J* = 8 Hz)/98.6	Liver, plasma
Glutamate	2.06 (m, CH_2_)/29.6; 2.36 (m, CH_2_)/36.1; 3.78 (m, CH)/56.7	Liver, bone marrow
Glutamine	2.16 (m, CH_2_)/29.1; 2.46 (m, CH_2_)/33.6; 3.78 (m, CH)/56.7	Liver, plasma, bone marrow
Glutathione (oxidized)	2.18 (m, CH_2_)/29.1; 2.55 (m, CH_2_)/34.2; 2.98 (m, CH)/41.7; 3.31 (m, CH)/41.6; 3.78 (m, CH_2_)/46.2; 3.78 (m, CH)/56.7	Liver, bone marrow
Glycerophosphocholine	3.22 (s, N(CH_3_)_3_)/56.9; 3.68 (m, NCH_2_)/68.9; 4.33 (m, CH_2_)/62.1	Liver, plasma, bone marrow
Glycine	3.56 (s, CH_2_)/44.3	Liver, plasma, bone marrow
Glycogen	3.66 (m, CH)/79.3; 3.98 (m, CH)/75.8; 5.41 (m, CH)/102.2;	Liver
Inosine	4.28 (m, CH)/88.2; 4.44 (m, CH)/73.1; 6.11 (d, *J* = 5.7 Hz, CH ring)/90.9; 8.23 (s, CH ring)/148.9; 8.32 (s, CH ring)/142.7	Liver
Isoleucine	0.93 (t, *J* = 7.2 Hz, CH_3_)/13.9; 0.99 (d, *J* = 7.2 Hz, CH_3_)/17.4	Liver, plasma
Lactate	1.33 (d, *J* = 7.2 Hz, CH_3_)/22.9; 4.11 (q, *J* = 7.2 Hz, CH)/71.2	Liver, plasma, bone marrow
Leucine	0.94 (d, *J* = 6 Hz, CH_3_)/23.6; 0.97 (d, *J* = 6 Hz, CH_3_)/24.7; 1.71 (m, CH)/27.3; 1.71 (m, CH_2_)/42.6	Liver, plasma
Lipids	0.88 (m, CH_3_); 1.30 (m, (CH_2_)_n_); 1.40 (m, CH_2_); 5.30 (m, CH = CH)	Plasma, bone marrow
Lipids (LDL, VLDL)	0.87 (m, CH_3_)/14.7; 1.28 (m, CH_2_)/34.6;	Plasma
Lysine	1.48 (m, CH_2_)/24.6; 1.72 (m, CH_2_)/29.1;1.91 (m, CH_2_)/32.7; 3.01 (m, CH_2_)/42.1	Liver, plasma, bone marrow
Methionine	2.12 (s, CH_3_); 2.18 (m, CH_2_); 2.64 (t, *J* = 7.2 Hz, CH_2_)	Plasma
Nicotinic acid	7.60 (dd, *J* = 7.9 and 5 Hz, CH ring); 8.25 (m, CH ring); 8.58 (m, CH ring); 8.92 (s, CH ring)	Liver
Phenylalanine	7.43 (t, *J* = 7 Hz, CH ring); 7.38 (t, *J* = 7 Hz, CH ring); 7.32 (d, *J* = 7 Hz, CH ring)	Liver, plasma, bone marrow
Phosphorylcholine	3.22 (s, N(CH_3_)_3_)/56.9; 3.60 (m, CH_2_)/69.4; 4.18 (m, CH_2_)/60.7	Liver, plasma, bone marrow
Succinate	2.41 (s)/36.8	Liver
Taurine	3.26 (t, *J* = 7.3 Hz, CH_2_)/49.9; 3.43 (t, *J* = 7.3 Hz, CH_2_)/38.1	Liver, plasma, bone marrow
Tyrosine	6.90 (m, CH ring); 7.18 (m, CH ring)	Liver, plasma, bone marrow
Uridine	4.14 (m, CH)/86.9; 4.38 (m, CH)/76.4; 5.88 (m, CH ring)/104.9; 5.90 (m, CH)/92.1; 7.88 (d, *J* = 8.10 Hz, CH ring)/144.5;	Liver
Valine	0.99 (d, *J* = 7 Hz,CH_3_)/19.5; 1.05 (d, *J* = 7 Hz,CH_3_)/20.7; 2.28 (m, CH)/31.9	Liver, plasma

**Table 2:** Intercept values (*R* ^²^/*Q* ^²^) of permutation test for the PLS-DA models.

PLS-DA model	Female				Male			
	Nonexposed		Exposed		Nonexposed		Exposed	
	*R* ^²^	*Q* ^²^	*R* ^²^	*Q* ^²^	*R* ^²^	*Q* ^²^	*R* ^²^	*Q* ^²^
Plasma	0.401	−0.303	0.402	−0.301	0.505	−0.406	0.508	−0.411
Bone marrow	0.581	−0.322	0.585	−0.352	0.912	−0.205	0.908	−0.158
Liver	0.077	−0.307	0.084	−0.316	0.181	−0.350	0.185	−0.346

The intercept values (*R* ^²^/*Q* ^²^) represent the values of *R* ^²^ and *Q* ^²^ of a purely random model. *R* ^²^ intercept <0.3 and *Q* ^²^ intercept <0.05 indicate a robust model.

**Table 3:** Summary of the relative metabolite changes in plasma, bone marrow, and liver upon long term dietary exposure of mice to low doses of endosulfan.

Major metabolites	Discriminant NMR buckets	Bone marrow		Plasma		Liver	
		Female	Male	Female	Male	Female	Male
Lipids VLDL/LDL	1.26; 1.30; 1.34; 1.38			↘**			
Lipids	0.90; 1.30; 1.34	↘***	↘*				
Unsaturated lipids	5.34	↘***					
Lactate	1.34; 4.10		↘*		↗*	↗**	↘**
Choline/PCho/GPCho	3.18; 3.22; 4.02; 4.22; 4.30	↗**	↘*	↘*			↗*
Glucose	3.26; 3.38; 3.42; 3.46; 3.50; 3.54; 3.82; 3.90; 5.22			↗***	↗*	↘***	
Glycogen	5.40						↘*
Lysine	1.66; 1.70; 1.74; 3.02	↗**					
Valine	0.98	↗*	↗*				
Isoleucine	0.94	↗**	↗*		↗*		
Alanine	1.46; 1.50	↗**					↘***
Glutamate/glutamine	2.10; 2.14; 2.34; 2.38; 2.46	↗**					↘***
Glutathione (oxidized)	2.18; 2.54; 2.58; 3.78					↘*	↘***
Betaine	3.26					↗***	↗***
Taurine	3.26; 3.42					↗***	↗***
Acetate	1.94		↗*				
Tyrosine	6.90; 7.18; 7.22		↗**				
Inosine	6.06; 8.22		↗**				
ATP/ADP/AMP	4.42; 4.38; 6.14; 8.26; 8.58		↘***				
Succinate	2.42	↗**	↗**				
Creatine	3.94		↗*				

Comparisons among groups were performed by Kruskal-Wallis test. Changes are relative to control samples: *↘*: decrease; *↗*: increase; ***P < 0.001; **P < 0.01; *P < 0.05 as compared with control.
